# Coordination of care in the context of primary brain tumors: Australian healthcare professionals’ exploration of the unspoken impact and clinical implications

**DOI:** 10.1007/s00520-026-11107-w

**Published:** 2026-08-27

**Authors:** Megan S. Jeon, Hannah Isaac, Joanne Shaw, Dianne M. Legge, Sharon He, Thomas Carlick, Eng-Siew Koh, Georgia K. B. Halkett, Brian Kelly, Mark B. Pinkham, Raymond J. Chan, Tamara Ownsworth, Haryana M. Dhillon

**Affiliations:** 1 Psycho-Oncology Co-Operative Research Group (PoCoG), School of Psychology, Faculty of Science, The University of Sydney, Sydney, Australia; 2 Oliva Newton-John Cancer and Wellness Centre, Austin Hospital, Heidelberg, VIC Australia; 3 Faculty of Health Sciences, Curtin School of Nursing, Curtin University, Perth, Australia; 4 Liverpool Cancer Centre, Liverpool Hospital, South Western Sydney Local Health District, Sydney, Australia; 5 South West Sydney Clinical School, University of New South Wales, Sydney, NSW Australia; 6 School of Medicine and Public Health, University of Newcastle (UON), Callaghan, NSW Australia; 7 Department of Radiation Oncology, Princess Alexandra Hospital, Woolloongabba, QLD Australia; 8 Caring Futures Institute, College of Nursing and Health Sciences, Flinders University, Bedford, Australia; 9 The Hopkins Centre, School of Applied Psychology, Griffith University, Nathan, QLD Australia

**Keywords:** Care coordination, Model of care, Brain cancer, Healthcare professionals, Survivorship

## Abstract

**Aim:**

People with primary brain tumor (PBT) and their families experience high distress and face challenges navigating their disease and treatment, healthcare systems, and/or survivorship. Quality brain tumor care coordination (BTCC) enables continuous, timely care according to the individual care needs of patients. We aimed to explore healthcare professionals’ (HCPs) practices and perceptions of BTCC.

**Methods:**

We conducted semi-structured interviews with 12 HCPs (92% female) working with people with PBT from Australia and Aotearoa New Zealand between June and November 2023. The median interview duration was 58 min. Qualitative data were analyzed thematically, with a framework matrix for data organization and systematic coding.

**Results:**

Participants had a median of 13 years of experience in neuro-oncology across medical, nursing, and allied health disciplines, and reported experience in BTCC. We identified four themes related to BTCC: (1) Vulnerable and complex: Pervasive, multifaceted disabilities of PBT require specialist care; (2) Tailored coordination: Shaping and tailoring BTCC is in the best interests of patients and families; (3) Going the extra mile: Care needs warrant proactive support to mitigate crises; and, (4) Emotionally demanding: HCPs manage emotional load with limited support.

**Conclusion:**

People with PBT experience a unique and complex set of care needs requiring specialist skills and knowledge beyond general oncology care coordination. BTCC encompasses continuous coordination of multidisciplinary care to optimize functioning for these individuals. Given the complexity of the BTCC role, better support infrastructure with training and resources, and appropriate interventions for HCPs are needed to retain HCPs and prevent burnout.

**Supplementary Information:**

The online version contains supplementary material available at https://doi.org/10.1007/s00520-026-11107-w.

## Importance of the study

Primary brain tumor treatment and survivorship present complex and unique challenges for patients and carers. Given the need for multidisciplinary involvement in management, access to quality care coordination is critical to obtaining timely, personally tailored care for people with primary brain tumor. Brain tumor care coordination plays an integral role in delivery of this care, but healthcare professionals carry a substantial emotional burden, placing them at risk of burnout if not supported. In this paper, we present in-depth, qualitative exploration of healthcare professionals’ perception of care coordination in the context of primary brain tumor, providing rich insights into the unique, nuanced clinical implications and impact on healthcare professionals working with this highly vulnerable population. 

## Introduction

Care coordination is the organization of care activities, communication, and collaboration among participants responsible for an individual’s care to facilitate delivery of services [1]. Cancer care coordination underpins comprehensive care as many people require multimodal, interdisciplinary treatment and support for comorbid conditions [2]. Fragmented, poorly coordinated care has been linked to poor symptom control, medical errors, and higher costs [1, 2]. Care coordination is key to delivering optimal care for primary brain tumor (PBT) [3, 4]. *Brain tumor coordination of care* (BTCC) is “a comprehensive approach to holistic person-centered healthcare and survivorship support for the duration of brain tumor experience according to the individual patient and family’s needs and preferences, through integration, collaboration, connectedness, and continuity across components of multi-disciplinary care, and timely delivery of services in an appropriate and complementary manner” (Box 1, page 13) [5].

Complexity of coordinating and delivering access to services from multiple providers is exacerbated for rare cancers [6, 7]. Malignant PBTs are rare and associated with poor prognosis and high morbidity and mortality rates [8, 9]. People with malignant PBT report diverse symptoms, poor emotional well-being, and high needs [10–14], requiring tailored, specialist and community-based care [15].

Models of BTCC rely on healthcare professionals (HCP) overseeing patient navigation, communication, education, coordination and referrals [16, 17]. However, BTCC varies based on geographical location, caseload, or care models. To address this need, a number of dedicated BTCC care coordinators (currently ~ 20–30) have been introduced in some Australian neuro-oncology services. The increase in BTCC care coordinators has been sporadic and uncoordinated, meaning more evidence is needed regarding what BTCC entails, including the nature, caseload, and impact of work and support available.

We aimed to explore HCPs’ current practices, the nature and impact of their experience with BTCC, and perceptions of PBT-specific implications for care coordination.

## Methods

### Study design

Reflexive thematic analysis to explore experiences of HCPs with BTCC for people with PBT [18–20]. The University of Sydney Human Research Ethics Committee (#2023/287) provided approval. Reporting adhered to consolidated criteria for reporting qualitative research (Supplementary File) [21].

### Participants

HCPs working with adults (≥ 18 years) with PBT were invited to participate via social media (‘X’ formerly twitter; LinkedIn), investigators’ networks, professional organizations, and passive snowballing.

HCPs were eligible if they were: (a) currently, or within five years, employed in a clinical or care coordinator role; (b) provided health services, support, or care for adults with PBT.

### Procedure

Invitations were distributed via channels described above. After consenting, participants provided demographic and professional and contact details. Interviews were conducted and audio-recorded using Zoom videoconferencing by MSJ, who had prior contact with study participants in professional settings. Interviews were transcribed using TRINT [22], transcripts were de-identified and checked by HB and SH. All investigators were experienced in clinical and qualitative research (Supplementary Material).

Our sample size was guided by information power [23]. Information power proposes the number of participants needed is influenced by information quality [23]. A sample size of 12 was deemed sufficient due to interview length, depth of information, and sample of highly knowledgeable and experienced HCPs in BTCC.

### Measures

A REDCap survey [24] captured demographic and professional characteristics. A semi-structured interview guide explored experiences of BTCC (Supplementary Material). This guide was iteratively developed by a stakeholder advisory group comprising HCPs, researchers, and consumers (people with lived experience of primary brain tumor or providing information care).

### Data analysis

SPSS [25] was used to analyze demographic data descriptively. Qualitative data were analyzed using a reflexive thematic analysis informed by elements of the framework approach for data organization and systematic coding [18, 19]. After transcription, MSJ, HB, DL familiarized themselves with the data. The interview guide was used to deductively develop preliminary ‘codes’. Researchers independently applied codes to three transcripts while inductively identifying additional codes to draw out patterns. Discrepancies were resolved via discussion and a comprehensive coding framework developed. HMD, GKBH reviewed the framework before application to transcripts using NVIVO [26].

## Results

### Demographic and clinical characteristics

Twelve HCPs caring for people with PBT participated. Interviews lasted a median 58 min. Most participants had median of 13.5 years clinical experience in neuro-oncology. Care delivery mode, clinical setting, and tumor types varied (Table 1).

**Table 1:** Demographic and clinical practice characteristics of participants (*N* = 12)

Demographic and clinical characteristics	*N*	%
Median Age, years	45 (IQR = 19)	
Gender		
• Female	11	92
Education		
• Bachelor	2	17
• Masters	7	58
• Doctorate/PhD	1	8
• Other^a^	2	17
Median years of clinical experience	16 (IQR = 17.3)	
Median years of clinical experience in neuro-oncology	13.5 (IQR = 8.5)	
Clinical qualification/background		
• Nursing	6	50
• Medical Oncology	3	25
• Neuroscience	1	8
• Neurosurgery	1	8
• Allied Health (Radiation therapy)	1	8
Current clinical role title		
• Nurse practitioner/Clinical nurse consultant/specialist	7	58
• Medical Oncologist	2	17
• Neurosurgeon	1	8
• Brain cancer/Neuro-oncology care coordinator	2	17
Main tumor type seen in clinical practice		
• High grade only	7	58
• Mixed: benign, low grade and high grade	5	42
Location of workplace		
• Metropolitan	12	100
• Rural/Regional	0	0
Location of workplace		
Australia (State)		
• New South Wales	5	42
• Victoria	3	25
• Australian Capital Territory	2	17
• Western Australia	1	8
Aotearoa New Zealand	1	8
Clinical setting ^b^		
• Tertiary cancer centers	10	83
• District/local public hospital—outpatient	4	33
• District/local public hospital—inpatient	2	17
• Private hospital	4	33
• Private cancer center/practice – outpatient	2	17
Care delivery mode^b^		
• Face-to-face in cancer center	12	100
• Telehealth/telephone consult	10	83
• Online meeting tool (e.g., Coviu or Zoom)	4	33
• General Practice Clinic	1	8
• Home visits	1	8

This table is replicated in the report of a parallel study conducted by the author group with the same sample ^a^Other included Bachelor + post-graduate and Diploma, post-grad cert; ^b^ Multiple answers possible

### Qualitative findings

We identified four themes, comprising ten subthemes (Table 2). Themes are described with accompanying quotes and participant identifiers, see Table 3 for additional quotes.

**Table 2:** Themes and subthemes

**Theme 1:** Vulnerable & complex: Pervasive, multifaceted disabilities of PBT require specialist care	**Theme 2:** Tailored coordination: Shaping and tailoring BTCC is in the best interest of people with PBT & their families	**Theme 3:** Going the extra mile: Care needs warrant proactive support to mitigate crises	**Theme 4:** Emotionally demanding: HCPs manage emotional load with limited support
1.1. The unique multifaceted disabilities of PBT	2.1. Fostering multidisciplinary collaboration & patient navigation	3.1. Proactive support	4.1. Emotionally demanding tasks
1.2. Rippling impacts on the patient as a person in their context	2.2. Person-centered coordination	3.2. Supporting the carers and family	4.2. Emotional skills and engagement
	2.3. Promoting wellness with illness		4.3. Emotional impact on HCPs

*PBT* Primary brain tumor, *BTCC* Brain tumor care coordination, *HCP* Healthcare professional

**Table 3:** Supportive quotes for themes and subthemes

Theme	Subtheme	Attribute	Quote
Theme 1 – Vulnerable and complex: Pervasive, multifaceted disabilities of PBT require specialist care	*1.1. The unique, multifaceted disabilities of PBT*	Unique cognitive deficits of PBT	“The cognitive disability associated with the disease, I think is quite unique in cancer; the patient's not necessarily their own advocate the entire time through their disease course.” P13 *“*Absolutely every single one of them [people with PBT] has cognitive impairment to some degree, and that changes everything.” P14
		Distressing nature of PBT symptoms for carers and family members	“Because it's impacting on the brain, depending on the location of the cancer, it can be quite significant, not just physically, but yeah, those behavioural cognition, or emotional aspects can be actually directly impacted by the cancer and can be challenging.” P3 “The family brings in a person with a personality. And after surgery, they're very likely to take from somebody who has greater needs or even has a changed personality.” P10
		General HCPs may not feel competent in dealing with unique PBT symptoms	“I find people who've never had experience with brain tumours often find it really like either confusing or, don't really know how to help.” P8
	*1.2. Rippling impacts on the patient as a person in their context*	Widespread impact of PBT	“It [PBT] comes out of the blue. These people are fit, they’re healthy, they’re young and, their whole function can be changed… their whole brain, the way they speak, the way they interact, their personality can change. Like it's a really big impact, quickly.” P9
		High level of carer burden	“We rely heavily on the carers, if there are no carers, it's a massive issue for us.” P6
		People with PBT described as a vulnerable group of patients	“I've worked with lots of different cancers. I've worked with sarcomas, predominantly younger people. But even then, there's hope. I feel like there's not as much hope when you get that [PBT] diagnosis.” P9 “It's not only the diagnosis and the treatment, it's actually the quality of life that's completely impacted as well.” P9
Theme 2 – Tailored coordination: Shaping and tailoring BTCC is in the best interest of people with PBT and their families	*General*	Perceptions of optimal BTCC	“The best care coordination would be to support the patients and the carers from the diagnosis until the end of life, basically. Being there, being able to provide support and services that would improve their quality of life or maintain the quality of life for the carers; for the patients, but also for the carers so that they, you know, aren't as burdened” P4
	*2.1. Fostering multidisciplinary collaboration and patient navigation*	Importance of coordinating multidisciplinary care	“We try we try to be one point of contact so that the person is not chasing things around in circles basically” P2
		Importance of clinical expertise	“The care of people with brain tumors tends to be a bit sub-specialized and…the ins and outs of management can be a little bit intimidating.” P13
	*2.2. Person-centered coordination*	Importance of tailored care	“It's something that needs to be tailored to each patient.” P12 “We don't have a strict structure that we follow or a specific pathway that we put a patient on. I mean, apart from, we know they need surgery and then they'll be offered chemo-radiation, but we're very much guided by their individual needs and their family's needs.” P2
		Importance of patient satisfaction	“I guess the quality indicators would probably be, look to me personally, would be like patient and family feedback. So I guess them letting you know that they feel supported.” P8
	*2.3. Promoting wellness with illness*	Importance of holistic care coordination	“It's not just about making appointments and navigating the system. It's about supporting their psychosocial needs, educating them, re-referring them, referring them to psych, rehab, fertility, all these other things as well.” P5
Theme 3 – Going the extra mile: Care needs warrant proactive support to mitigate crises	*3.1. Proactive support*	Importance of triaging and staying on top of patient symptoms to avoid medical crises	“I guess it's just managing that in a timely manner to try to prevent deterioration.” P2 “A big mark of me achieving what I do is they're [patients] not bouncing into hospital every 5 min and you're keeping them out… If you could prevent, you know, readmission to hospital, for one, it's a big benefit for the health care dollar. But, from a psychological perspective, the patient is better because they're not coming in through emergency.” P5
		Taking on additional tasks eases patient loads but adds to participants’ own workloads	“I usually go see the patient and I actually sit in on the consult. That's not something that every cancer coordinator does… but most of the time they actually want me there.” P8 “Well, we [co-worker] end up calling each other after work, which obviously eats into home life… We do an email dump at the end of each shift. But that can sometimes make us late because there's lots of information you need to get in there. So we're constantly leaving work late to try and communicate between each other.” P12
	*3.2. Supporting the carers and family*	Carer support inherent to BTCC role	“A lot of it is really empowering and engaging with the caregiver, because they're the main one that's really overseeing what's going on.” P5 “I would say over 50% is supporting carers… there's a lot of carer distress and fatigue that is associated with brain tumours.” P8
		Importance of supportive care for carers when patient is reaching end-of-life	“As it starts to get towards the end of the patient journey, it's mostly the caregivers that I support.” P2
Theme 4 – Emotionally demanding: HCPs manage emotional load with limited support	*4.1. Emotionally demanding tasks*	Importance of psychological care in BTCC	“I've probably learned that it's a lot of, especially in this tumour stream, a lot of psychological counselling, that sort of stuff, the clinical psych sort of stuff.” P5 “You have to have a lot of difficult conversations with people because people can deteriorate quickly or things will change. And it's hard reminding someone, you know, that there's sometimes stuff we can't do to reverse this.” P9
		Distress and emotional load of BTCC sometimes unexpected	“You're kidding yourself if you don't think that you're going to be dealing with a lot of grief and a lot of kind of transitioning to end-of-life care and that sort of stuff.” P13
	*4.2. Emotional skills and engagement*	Importance of emotional intelligence and interpersonal skills	“It's a real skill set to catch that emotional cue, even though it's not verbally expressed. Yeah, and providing support, especially when the patient doesn't have any support.” P12
		Importance of not being too emotionally involved	“I'm a bit of a sensitive “Sally” as well, so I often, if people are upset and I can feel that, then I often have to have a cry after because it is terribly sad.” P8
	*4.3. Emotional impact on HCPs*	Emotional impact of BTCC	“You've just exhausted emotionally by the end of the day, most of the time. Because invariably there would be someone that you'd have to just absorb all of this sadness, anger, whatever. It can be quite personal.” P13
		Higher emotional impact than other tumor streams	“I’d say it’s a high burnout area.” P5
		Self-management strategies	“You can't make everybody well. And you've got to remember that when you go into this job. No matter what you do, that person is still going to die.” P4 “I'm lucky to have good relationships with the team that I work with, the doctors and the allied health are just a really good team. So I guess it's a lot of informal support that we all draw on.” P2

*BTCC* Brain tumor care coordination, *PBT* primary brain tumor, *HCP* healthcare professional

**Theme 1** Vulnerable and complex: Pervasive, multifaceted disabilities of PBT require specialist care.

#### Unique, multifaceted disabilities of PBT

Participants highlighted PBT is *“basically a brain injury”* [P9] with unique, multifaceted impacts on cognition, physical abilities, and personality. Participants reported universal and all-encompassing cognitive deficits create complex challenges. For example, comprehension, communication, and memory deficits manifest as irrational, inappropriate, or risky behaviors with legal implications, complicating care as patients were often unable to navigate health systems, comprehend treatment plans, or access care.

*“I think the main difference to other cancer care coordination is the actual neurological symptoms that are debilitating and how they impact on the patient.” [P3]*

Participants highlighted that, depending on tumor location, patients can experience physical disabilities (e.g., seizures) and personality changes (e.g., rigid thinking). These changes are highly distressing for patients, carers, and family.

*“It's the cognitive changes, memory, personality changes. I think they're the areas that are so hard for the carers and caregivers and family to understand.” [P5]*

Several participants suggested general HCPs may not feel competent dealing with these *“intimidating”* [P13] symptoms, emphasizing the need for specialist expertise.

*“There are unique issues to brain cancer that oncologists that don't treat brain cancer might not be as comfortable with” [P13]*

#### Rippling impacts on the patient as a person in their context

Participants noted the physical, cognitive, and personality changes seen in PBT create complex rippling impacts on every aspect of life, including finances, work, legal authority, relationships, independence, and mental wellbeing.

*“It [PBT] just touches so many aspects of people's lives, like it's just so pervasive” [P2]*

Informal carers were described as essential care partners due to high needs among PBT patients and their roles in decision-making. However, several acknowledged the high burden, distress, and need among carers.

*“I think the carers would be more burdened than most of the cancer types... and that's just because of all the other cognitive, personality and mobility issues. The fact that they can't drive, that their memory's poor, all those things that you don't get with a lot of other cancers” [P4]*

Participants highlighted psychological challenges faced by PBT patients and carers, due to the poor prognosis and *“out of the blue”* [P9] diagnosis, culminating in perceptions people with PBT are *“vulnerable”* [P12] and require specialist care.

*“Some people are just so vulnerable … they haven't got it in them to fight…or they've lost whatever fight they did have. It's been taken over by a brain tumor and they're just broken people.” [P12]*

**Theme 2** Tailored coordination: Shaping and tailoring BTCC is in the best interest of people with PBT and their families.

Many participants perceived autonomy and flexibility in their roles. While there was no one model of care coordination and approaches varied, participants agreed optimal BTCC should provide timely, continuous, and tailored multidisciplinary care across the disease continuum to prevent patients from *“falling through the cracks”* [P6] and achieve *“wellness with illness”* [P14].

*“That’s what our whole job is all about, making sure that patients receive the type of care that they need and they receive it when they should receive it and that they don’t miss out on anything.” [P6]*

#### Fostering multidisciplinary collaboration and patient navigation

While specific approaches to BTCC varied, many perceived themselves as a *“bridge person”* [P10] helping patients navigate complex health systems. Others described themselves as a *“jack of all trades”* [P9] fostering multidisciplinary collaboration or the *“glue that holds”* [P8] MDTs together for patients.

*“I kind of see myself as a bit of a navigator role as well. I think when people have diagnoses and they share so many different treating teams, that can be really confusing. So I’m kind of the glue that holds all these teams together for patients.” [P8]*

While these concepts apply to general cancer coordination, many described BTCC as *“quite different to care coordination of other cancers”* [P10] due to the complexity of symptoms and MDTs involved. The need for specialist training was emphasized as most participants were *“primary contacts”* [P3/P5] offering clinical consultations, expertise, and education to patients, carers, and other HCPs.

*“Ultimately, what the role is about is making the journey through the healthcare system much easier for the patient and the caregiver. And being one point of contact. And being, for me, it’s about being an expert in that field.” [P5]*

#### Person-centered coordination

Several participants noted care coordination is *“not just about making appointments and navigating the system”* [P5], but tailoring coordination to address unmet needs. The complexity of PBT impacts and needs, meant most participants felt care coordination should provide tailored, person-centered support guided by *“what works best for people”* [P9]. This included clinical consultations, education, psychological support, referrals, problem-solving, needs assessments, and care planning.

*“It has to be structured, but it has to be flexible to a degree…It’s tailoring the needs of the patients and the families in a way that is professional, but caring” [P12]*

Good communication, interpersonal, and patient advocacy skills were key to fostering safety, respect, and support. Many participants stated *“patient satisfaction is key”* [P3], using feedback as an indicator of care quality.

*“The most important thing is the person and their family feeling supported and feeling like their priorities are being heard and respected … that’s number one.” [P2]*

#### Promoting wellness with illness

Due to PBTs’ poor prognosis, participants emphasized the importance of shortening the period of ill health through support, particularly in the context of providing tailored support and referrals to manage physical and psychological disease impacts. This holistic approach benefits patients, carers, and healthcare systems by reducing hospitalizations.

*“I think good care coordination keeps people out of health care and, tries to promote wellness with illness.” [P14]*

**Theme 3** Going the extra mile: Care needs warrant proactive support to mitigate crises.

#### Proactive support

Flexibility in roles, combined with the devastating and time-critical nature of PBT, led participants to provide proactive, support beyond their prescribed duties. Many reported going the extra mile in caring, triaging needs, and managing risks to *“prevent deterioration”* [P2] and avoid crises, hospitalizations, and carer burnout.

*“You’re firefighting a little bit… It’s lots of little fires that you're putting out and managing as you’re going along to try and make that person’s journey through the treatment as easy as it can be.” [P5]*

These tasks did not cross professional boundaries but were extensions of care coordination duties. This included arranging at-home community support, preparing materials for social services (in Australia, Centrelink, National Disability Insurance Scheme [NDIS]), after-hours debriefing and handover, and attending appointments to provide support and advocacy. While this eased the load on patients, carers, and other HCPs, it increased participants’ own workload with no recognition of need for greater resources.

*“A lot of paperwork like Centrelink and NDIS, that paperwork, like that's ridiculous… a lot of my patients look at it and just say it’s too much. So I guess that’s an extra aspect that we try to absorb for them.” [P2]*

#### Supporting carers and family

Most participants acknowledged the role and experiences of carers in PBT, describing them as *“an extension of the patient”* [P4] or *“the most significant person in the whole picture”* [P14]. Despite the lack of funding to facilitate support to carers, most participants felt it inherent to their role.

*“If the carer feels more comfortable, feels like someone’s listening to them too, then they’ll deliver better care. So, it’s part of the job” [P9]*

This included *“empowering and engaging”* [P5] carers through upskilling, *“emotional support and information sharing”* [P14]. This was especially important for those caring for someone with functional deficits, personality changes, or at *“the end of the patient journey”* [P2].

*“That’s part of your care coordination; it’s trying to help carers at least to cope with how to look after these people.” [P10]*

*“When the person stops having insight into how unwell they are or they feel like they can’t manage it anymore that’s when you almost see it flip and, you’re caring mostly for the carer.” [P2]*

Contrastingly, some participants shared they can *“only do so much”* [P4] for carers due to workload and patient-centric healthcare systems.

**Theme 4** Emotionally demanding: HCPs manage emotional load with limited support.

#### Emotionally demanding tasks

Due to the devastating impacts and prognosis of malignant PBT, participants commented on the importance of providing psychological support and care.

*“It’s terminal more or less from day one. So there’s all of those psychological things … supporting people who are dying basically.” [P4]*

HCPs provided psychological assistance while managing raw emotions, *“difficult conversations”* [P9] and uniquely distressing PBT side effects. Effective BTCC navigates these situations with empathy, reassurance, and compassion while *“balancing hope and realism”* [P13]. While most participants perceived these *“emotionally taxing”* [P13] tasks as *“part of the job”* [P13], some were surprised by the depth of emotional weight.

*“I probably wasn’t aware of how much distress I would be dealing with…a lot of it is very heavy and very sad.” [P8]*

#### Emotional skills and engagement

Most participants viewed emotional intelligence and highly developed interpersonal skills as requisite for quality BTCC. These skills are essential in responding to emotionally laden situations in a professional but empathetic manner; advocating for patients’ needs; recognizing emotional cues; managing expectations; and building connection, trust and relationships.

*“I think high-quality care coordination would be … someone who is trustworthy, someone who is reliable, someone who is really good at communicating” [P8]*

Some noted a degree of emotional involvement. However, it was a fine line to navigate — as being too emotionally involved may hinder delivery of quality care.

*“You need to be emotionally involved, but you also don’t want to get too overwhelmed because you’re going to be seeing a lot of these patients every day.” [P3]*

#### Emotional impact on HCPs

Despite the volume of emotional support provided to patients, participants were left to *“absorb”* [P13] the emotional load of these experiences and tasks *“every day basically that you're at work.”* [P4] with limited psychological support. Leaving them at risk of *“burnout”* [P5], distress, ruminating, redirecting patient/carer feelings and reactions to themselves (transference), personalization (e.g., imagining themselves in specific situations), and being *“exhausted emotionally”* [P13]. While these challenges are present in other cancer streams, they are especially evident in BTCC due to the pervasive impacts, high needs, and poor prognosis.

*“I often find myself renumerating [sic], like conversations I’ve had or … difficult things that have happened, at home. So I have found that I am taking a lot of, not actual work home, but just like the contents of what happened at work. I think I do that a lot.” [P8]*

*“I feel maybe brain tumor patients are just a little bit different in terms of how emotionally difficult it can be for the nurse, like being that point of contact all the time”. [P8]*

Importantly, no participant reported formal psychological support (e.g., debriefing) in their workplace. Participants implemented self-management strategies to mitigate the emotional impacts of their role. This included self-care, informal debriefing with colleagues, setting boundaries, compartmentalization, and mindset shifts (e.g., *“If I wasn’t here, imagine how worse off their journey could be”* [P9]). All strategies were self-directed and implemented outside work hours.

*“I’ve been doing it for a long time, so I try to distance myself to some degree … I provide care, but then I can compartmentalize… you need to be able to have that disconnect between work and home life” [P4]*

## Discussion

**Fig. 1:**
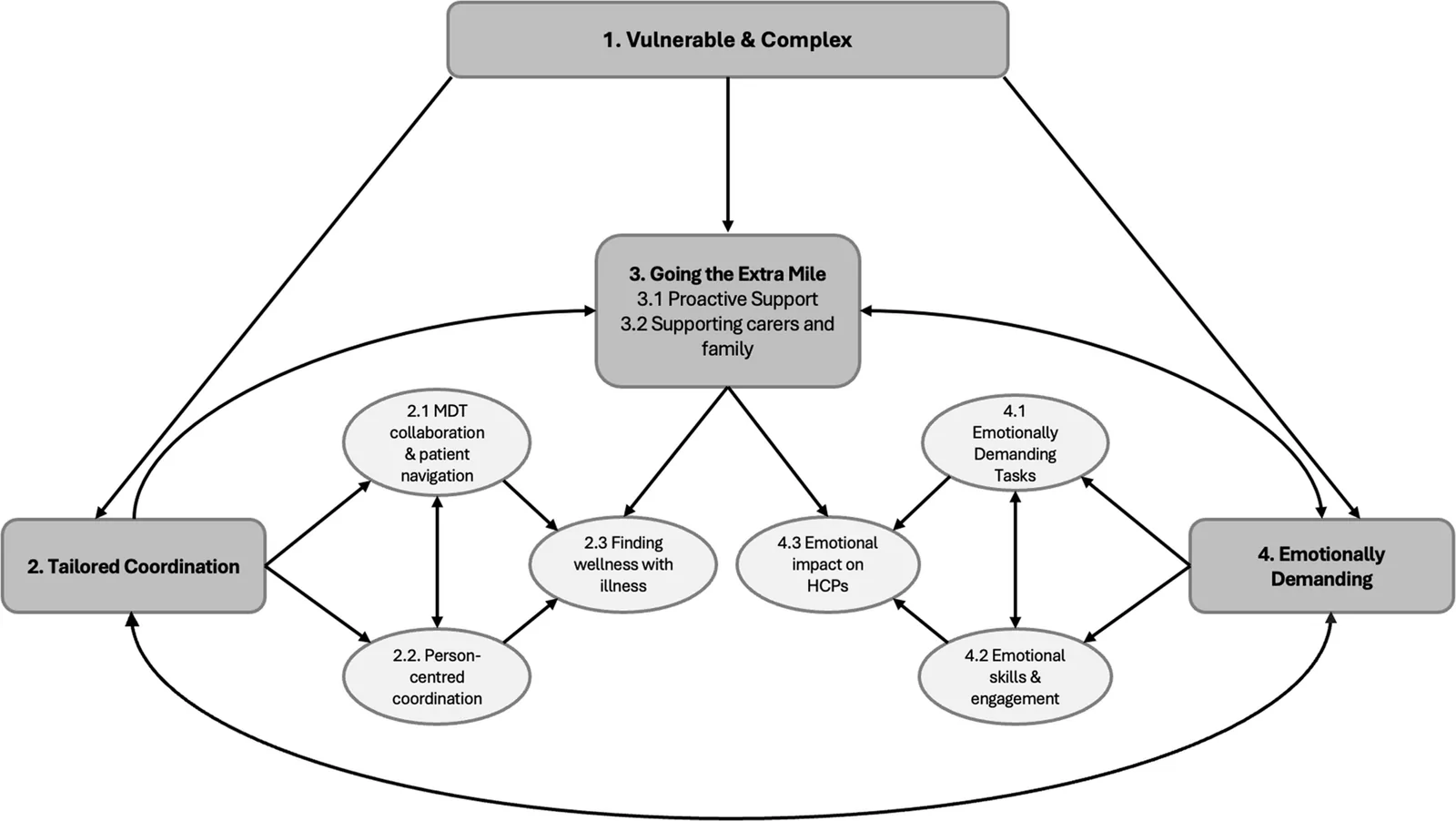
A conceptual model of brain tumor care coordination as perceived by healthcare professionals caring for people with primary brain tumor and their carers

We provide an in-depth, qualitative exploration of HCPs’ perception and approaches to BTCC, clinical implications of PBT for care coordination and emotional impact on HCPs. Based on the four themes identified, we developed a conceptual model to represent the interplay between key themes and their contribution to our understanding of BTCC (Fig. 1).

### Current practice of BTCC

The model depicts PBT-related multifaceted disabilities and pervasive impacts on individuals and their families (Theme 1) that underpin and shape key aspects of BTCC. HCPs take a flexible approach to BTCC, so the care plan and delivery is tailored to the diverse and evolving needs of individuals with PBT (Theme 2). They are key to two main components: multidisciplinary care and navigation (2.1) and person-centered coordination (2.2), both reciprocally impacting each other through communication, patient advocacy, and acting as a *“bridge person”*, to maximize wellness through quality care and support (2.3). The vulnerable, complex, and time-critical nature of PBT care leads HCPs to go the extra mile (Theme 3) beyond coordination of hospital-based care to proactively facilitate access to community-based care and services and support carers/family to prevent crisis. While this maximizes wellness with illness (2.3), additional supportive duties and work hours add to the emotional impact on these HCPs (4.3). Lastly, the nature and care needs of PBT make BTCC emotionally demanding (Theme 4). HCPs face emotionally demanding tasks (4.1) “almost every day at work”, managing intense emotional loads on patients and themselves. HCPs perceive emotional skills and engagement as necessary to provide appropriate support (4.2), which reciprocally impacts their perception of roles/tasks for delivering optimal, coordinated care (Themes 2 and 3), although this may compound the emotional impact on HCPs and their risk of burnout with limited support (4.3).

### Clinical implications for BTCC

HCPs highlighted the uniqueness of BTCC, attributed to the pervasive and multifaceted impact of the disease and its treatment, complexity of care required, and systemic and patient-level barriers to delivering care. People with PBTs often experience sudden onset of debilitating symptoms (e.g., seizures, headache), followed by rapid transition into diagnosis and treatment [7, 27]. The complexity of BTCC arises from the need for a large multidisciplinary, specialist care team. Past research similarly showed people with PBT experience a diverse range of physical (e.g., impaired coordination, mobility), cognitive (e.g., memory, communication), emotional and behavioral (e.g., agitation, distress), or neurological impairments (e.g., seizures, vision), some of which are persistent and irreversible [10, 11]. Long-term disability and tumor progression with functional decline are common [13, 28, 29]. BTCC should facilitate proactive assessment of needs and access to care and services tailored to individual care and support needs.

HCPs emphasized the nuanced and unique impact of PBT on personality, cognition and behavior, including adverse psychiatric reactions to treatment. HCPs noted PBT-induced personality and behavior changes may be “*intimidating*” to generalist care coordinators. These changes can present as aggression, apathy, paranoia, disinhibition, or emotional lability [30], and may evolve or emerge rapidly [31]. This places significant burden on carers, which is frequently underrecognized [32]. The lack of information and limited services present systemic challenges to supporting people with PBT and families [31]. HCPs must often build trust and rapport and to educate carers [33], consistent with our findings where HCPs overseeing BTCC *“put out small fires”* navigating emergent issues.

Cognitive changes generate substantial emotional and functional impact on patients and families, rippling across all life domains, but most critically, the patient’s sense of identity, autonomy, and safety. These challenges can reduce patients’ capacity to engage with healthcare systems [34]. This contributes to HCPs’ perceptions that this population are especially vulnerable and HCP perceived role in advocacy on the patients’ behalf.

The importance of including and supporting carers and family members in PBT care was central to achieving quality BTCC, even when creating additional work, which is unrecognized within the system. These perceptions reflect those of the broader neuro-oncology community view of what constitutes BTCC including carer recognition, assessment of needs, and support [5, 35].

### Emotional impact of BTCC on HCPs

Our findings demonstrated emotional and interpersonal demands of BTCC place a significant burden on these HCPs, making the risk of burnout concerning. HCPs highlighted their roles in BTCC require advanced emotional skills, engagement, interpersonal and communication skills, to navigate emotionally charged situations. Although emotional exhaustion and burnout are common among oncology professionals [36, 37], some of our sample reported the intensity and frequency of these interactions as part of their work surprising, given the depth of distress and emotional weight in PBT. HCPs delivering BTCC are often the sole designated primary contact, with little support and flexibility for time away from work, compounding risk of burnout [38, 39]. To sustain this workforce and ensure high-quality PBT care, improved support structures are warranted at both individual and institutional levels, fostering supportive community through clinical supervision and peer support, fair workload, training, and recognition of the emotional labor of BTCC [40].

### Limitations

This study has several limitations. No generalist HCPs participated, and the sample was from metropolitan cancer centers in Australia, limiting generalizability to regional settings. Due to duplicate memberships across organizations and snowballing recruitment, we were unable to calculate the response rate. The emotional impact of BTCC on HCPs working in regional areas may be underrepresented, although participants reported insolation within their institution. Additionally, our sample included few non-nurse, restricting diversity of perspectives.

## Conclusions

This study provided rich insight into the current approach to BTCC in Australia, its clinical and emotional implications for HCPs involved. BTCC involves continuous coordination of multidisciplinary person-centered care to optimize functional living for these individuals. The emotional demand of BTCC place considerable pressure on these specialist HCPs, increasing risk of burnout. To support the workforce and sustainability of BTCC, there is a need for robust support infrastructure, including training, adequate resourcing, and institutionally supported interventions addressing individual and systemic challenges.

## Supplementary Information

Below is the link to the electronic supplementary material.

Supplementary file 1 (DOCX 27 KB)

Supplementary file 2 (DOCX 24 KB)

## Data Availability

No data other than the deidentified aggregated group data presented in the manuscript will be made available to a third party in line with the ethical guidelines and conditions outlined in the participant consent.
