# Supplementary material for: Coordination of care in the context of primary brain tumors: Australian healthcare professionals’ exploration of the unspoken impact and clinical implications
Source: Support Care Cancer. 2026 Aug 27;34(9):901. doi: 10.1007/s00520-026-11107-w (PMC13521993; doi:10.1007/s00520-026-11107-w)
Supplement: Supplementary file 1 [file 520_2026_11107_MOESM1_ESM.docx]

SUPPLEMENTARY FILE

Completed COREQ Checklist

| **Topic** | **Item No.** | **Guide Questions/Description** | **Reported on**  **Page No.** | |
| --- | --- | --- | --- | --- |
| **Domain 1: Research team**  **and reﬂexivity** | | | | |
| *Personal characteristics* | | | | |
| Interviewer/facilitator | 1 | Which author/s conducted the interview or focus group? | 7 | |
| Credentials | 2 | What were the researcher’s credentials? E.g., PhD, MD | Supplementary Materials | |
| Occupation | 3 | What was their occupation at the time of the study? | Supplementary Materials | |
| Gender | 4 | Was the researcher male or female? | Supplementary Materials | |
| Experience and training | 5 | What experience or training did the researcher have? | 7 & Supplementary Materials | |
| *Relationship with*  *participants* | | | | |
| Relationship established | 6 | Was a relationship established prior to study commencement? | 7 | |
| Participant knowledge of  the interviewer | 7 | What did the participants know about the researcher? e.g., personal  goals, reasons for doing the research | 7 | |
| Interviewer characteristics | 8 | What characteristics were reported about the interviewer/facilitator?  e.g., Bias, assumptions, reasons and interests in the research topic | Supplementary Materials | |
| **Domain 2: Study design** | | | | |
| *Theoretical framework* | | | | |
| Methodological orientation and Theory | 9 | What methodological orientation was stated to underpin the study? e.g., grounded theory, discourse analysis, ethnography, phenomenology,  content analysis | 7 | |
| *Participant selection* | | | | |
| Sampling | 10 | How were participants selected? e.g., purposive, convenience,  consecutive, snowball | 7 | |
| Method of approach | 11 | How were participants approached? e.g., face-to-face, telephone, mail,  email | 7 | |
| Sample size | 12 | How many participants were in the study? | 9 | |
| Non-participation | 13 | How many people refused to participate or dropped out? Reasons? | NA – no drop outs | |
| *Setting* | | | | |
| Setting of data collection | 14 | Where was the data collected? e.g., home, clinic, workplace | 7 | |
| Presence of non-  participants | 15 | Was anyone else present besides the participants and researchers? | 9 | |
| Description of sample | 16 | What are the important characteristics of the sample? e.g., demographic data, date | 9 & table 1 | |
| *Data collection* | | | | |
| Interview guide | 17 | Were questions, prompts, guides provided by the authors? Was it pilot tested? | 8 | |
| Repeat interviews | 18 | Were repeat interviews carried out? If yes, how many? | NA | |
| Audio/visual recording | 19 | Did the research use audio or visual recording to collect the data? | 8 | |
| Field notes | 20 | Were field notes made during and/or after the interview or focus group? | NA | |
| Duration | 21 | What was the duration of the interviews or focus group? | 9 | |
| Data saturation | 22 | Was data saturation discussed? | 8 | |
| Transcripts returned | 23 | Were transcripts returned to participants for comment and/or correction? | NA | |
| **Domain 3: analysis and**  **ﬁndings** | | | | |
| *Data analysis* | | | | |
| Number of data coders | 24 | How many data coders coded the data? | | 8 |
| Description of the coding  tree | 25 | Did the authors provide a description of the coding tree? | | 8 |
| Derivation of themes | 26 | Were themes identified in advance or derived from the data? | | 8 |
| Software | 27 | What software, if applicable, was used to manage the data? | | 8 |
| Participant checking | 28 | Did participants provide feedback on the findings? | | NA |
| *Reporting* | | | | |
| Quotations presented | 29 | Were participant quotations presented to illustrate the themes/findings?  Was each quotation identified? e.g., participant number | | 11-19 & Table 3 |
| Data and findings consistent | 30 | Was there consistency between the data presented and the findings? | | 11-19 |
| Clarity of major themes | 31 | Were major themes clearly presented in the findings? | | 11-19, 20, Figure 1 |
| Clarity of minor themes | 32 | Is there a description of diverse cases or a discussion of minor themes? | | NA |
