# Supplementary material for: Coordination of care in the context of primary brain tumors: Australian healthcare professionals’ exploration of the unspoken impact and clinical implications
Source: Support Care Cancer. 2026 Aug 27;34(9):901. doi: 10.1007/s00520-026-11107-w (PMC13521993; doi:10.1007/s00520-026-11107-w)
Supplement: Supplementary file 2 [file 520_2026_11107_MOESM2_ESM.docx]

**Supplementary materials**

**A. Interview schedule**

Interviews and focus groups will be guided by a semi-structured interview schedule. Questions will not be asked verbatim but will provide guidance within the main theme to be explored. Topics discussed using the interview schedule will include, but are not limited to:

1. **Background information on the clinical practice setting and roles in care coordination**
   - Can you briefly describe your practice setting and your role in care coordination for people with primary brain tumours?
   - What is care coordination in your opinion?
   - What does effective care coordination look like in your practice and what indicates if it is effective? Can you give an example?
2. **Informational, educational, and professional training needs of HCP involved in care coordination for people with PBT and their family caregivers**
   - Have you received any formal training for your current role in care coordination for this group of patients?
     - If yes, what was it and how did you find the training program?
     - If no, what would you wish you knew before starting your current role if you went back in time?
   - In terms of education and professional training, what do you know about existing information or education resources that can be useful for someone new to the job in brain tumour care coordination?
   - What are some of the challenges when communicating with this group of patients and their carers?
     - What are the more difficult topics to discuss during your interactions?
     - What makes these topics difficult?
   - What area or topics would you like to have more opportunities to learn or upskill in?
   - What is your preferred method of accessing education and training support? Why?
   - What do you think are potential barriers to completing training, online or on-site?
3. **HCP’s perception of core and unique skill sets required for PBT care coordination and ways to engage HCP in general cancer care**
   - What is different or the same about PBT compared to general cancer care coordination? (what are the extra layers to consider for this cancer patient group?)
   - Are there any other challenges or barriers that you are aware of?
     - How about personality or behaviour changes or cognitive symptoms?
   - What training or support is needed for general cancer nurses, coordinators or other HCP to be involved in PBT care coordination?
4. **Need for other support, including social support, psychological, emotional or physical support or support for work-related stress and burnout**
   - How do you feel about other forms of support that HCP may require for their role in care coordination? They may include social support, psychological, emotional or physical support or support for work-related stress and burnout.
   - What do you think needs to be addressed to best support HCP involved in care coordination for people with PBT?
   - Do you feel you are able to provide support to the caregivers? Why or why not?
   - Are you aware of any formal or informal support that is previously or currently available within your practice or externally?
5. **Practical, resourcing, or logistical needs of HCP for communication and integration of care processes and referrals across sectors**
   - How do you go about contacting patients/carers at various stages and making referrals to community-based services?
   - What is the biggest challenge to providing continuous and connected care across sectors?
   - What will make it easier for you to communicate patient and caregiver needs or symptoms with other professionals outside your practice setting?
   - If you can have or change anything to help improve the follow-up and re-assessment process, what would it be?

**B. Research team and reflexivity**

Personal characteristics including credentials, occupation and experience:

DL – OT, professional experience in care coordination, PhD candidate experienced in qualitative research methods;

MJ – clinical psychology trained, research focusing on CC, with interest and background knowledge in the research topic gained from literature review and Delphi study running in parallel; experienced in qualitative and mixed methods research;

HB – RA, psychology trained, PhD candidate experienced in qualitative research methods;

HD – Prof, psycho-oncology researcher with more than 30 years experience in clinical and cancer research; experienced in qualitative and mixed methods research; and,

GH – Prof, radiation therapy trained, with more than 15 years experience in clinical and cancer research; experienced in qualitative and mixed methods research;.

Note. All authors who were involved in interview or data analysis were female and had no established relationship with participants.
